# Commentary: Heparin Attenuates Histone-Mediated Cytotoxicity in Septic Acute Kidney Injury

**DOI:** 10.3389/fmed.2021.647741

**Published:** 2021-04-20

**Authors:** Yupei Li, Yiran Wang, Luojia Jiang, Tao Song, Baihai Su

**Affiliations:** ^1^Department of Nephrology of West China Hospital, Institute for Disaster Management and Reconstruction, Sichuan University, Chengdu, China; ^2^State Key Laboratory of Polymer Materials Engineering, College of Polymer Science and Engineering, Sichuan University, Chengdu, China

**Keywords:** heparin, histone, coagulation, bleeding, septic acute kidney injury

We read with great interest the article entitled “Heparin Attenuates Histone-Mediated Cytotoxicity in Septic Acute Kidney Injury” by Wang et al. recently published in *Frontiers in Medicine* (1). In this article, the authors demonstrated that mice with cecal ligation and puncture- (CLP-) induced spetic acute kidney injury benefited from intraperitoneal heparin administration with improved survival, declined serum pro-inflammatory cytokines (namely tumor necrosis factor-a and interleukin-6) as well as kidney injury biomarkers (namely neutrophil gelatinase-associated lipocalin and kidney injury molecule-1), and decreased protein and mRNA expression levels of kidney apoptosis-related proteins (cleaved Caspase-3/Caspase-3 and Bax/Bcl-2) compared with those in the sepsis group at 6 h after CLP. Heparin may alleviate apoptosis and inflammation by neutralizing extracelluar histones and thus play a protective role against septic acute kidney injury.

Earlier literatures also showed that heparin and heparinoids could reduce histone-induced endothelial damage, inflammatory responses, coagulation activation, organ dysfunction and improve survival in septic mice (2–6). However, routine use of heparin in spetic patients is theoretically associated with high risk of fatal bleeding events as septic patients often develop disseminated intravascular coagulation (DIC) with excessive consumption of clotting factors and platelets (7, 8). Safety of direct heparin administration in patients with severe sepsis thus remains as a great concern. Unfortunately, the coagulation parameters as well as bleeding events in CLP-induced septic mice treated by 3 mg/kg of heparin were not available in the present work, preventing a more widespread clinical use of heparin as an anti-histone agent in patients with septic acute kidney injury.

Recently, non-anticoagulant heparins and small polyanions have been developed for histone neutralization to eliminate the bleeding concern of heparin use (6, 9). Wildhagen et al. demonstrated that an anti-thrombin affinity depleted heparin (AADH) could directly bind to histones with an apparent dissociation constant of 86 nM and effectively block histone-mediated cytotoxicity (6). Use of AADH in CLP-induced and lipopolysaccharide-induced septic mice significantly improved the survival rate and decreased neutrophil influx, intrapulmonary protein leakage and capillary-alveolar leakage with negligible prolongation of tail bleeding time (6). Likewise, O' Meara et al. developed non-anticoagulant O-sulfated small polyanions, derived from D-cellobiose, which interact electrostatically with histones and neutralize histone-mediated cytotoxicity, platelet aggregation and degranulation, and erythrocyte fragility (9). *In vivo* experiments further showed that these small polyanions could significantly inhibit histone-induced organ dysfunction, thrombocytopenia, anemia, and deep vein thrombosis as effective as heparin. However, at least 50 percent of mice treated by 6.25 mg/kg of heparin had to be euthanized due to bleeding in this study, highlighting the safety concern of heparin use in septic patients with DIC (9). Therefore, we recommend that further exploration should be performed to determine the potential advantage of non-anticoagulant heparins and other polyanions against unfractionated- or low-molecule-weight heparin in septic acute kidney injury in future.

## Author Contributions

YL and YW drafted the manuscript. All authors contributed to reviewing and revising the manuscript. All authors have given approval to the final version of the manuscript.

## Conflict of Interest

The authors declare that the research was conducted in the absence of any commercial or financial relationships that could be construed as a potential conflict of interest.
